# Improving prescribing: a feasibility study of pharmacogenetic testing with clinical decision support in primary healthcare in Singapore

**DOI:** 10.1093/fampra/cmac124

**Published:** 2022-11-23

**Authors:** Helen Smith, Martin Dawes, Hagit Katzov-Eckert, Sarah Burrell, Sam Xin Hui, Michael D Winther

**Affiliations:** Family Medicine and Primary Care, Lee Kong Chian School of Medicine, Nanyang Technological University, Singapore, Singapore; Department of Family Practice, The University of British Columbia, Vancouver, BC, Canada; GenXys Health Care Systems, Vancouver, BC, Canada; Family Medicine and Primary Care, Lee Kong Chian School of Medicine, Nanyang Technological University, Singapore, Singapore; Family Medicine and Primary Care, Lee Kong Chian School of Medicine, Nanyang Technological University, Singapore, Singapore; Family Medicine and Primary Care, Lee Kong Chian School of Medicine, Nanyang Technological University, Singapore, Singapore

**Keywords:** clinical decision support software, pharmacogenetics, primary care, Singapore

## Abstract

**Background:**

The study of genetic variation as a factor influencing drug safety, efficacy, and effectiveness has brought about significant breakthroughs in understanding the clinical application of gene–drug interactions to better manage drug therapy.

**Objective:**

This study was designed to assess the feasibility of collecting buccal samples by general practitioners (GPs) at private practices in Singapore within a usual consultation, incorporating use of a pharmacogenetics-based medical decision support system to guide subsequent drug dosing.

**Methods:**

We used a prospective cohort study design, with GPs recruiting 189 patients between October 2020 and March 2021. The genotypes of 51 biallelic SNPs were determined using Illumina Infinium Global Screening Array.

**Results:**

Seven GPs from 6 private practices recruited and obtained buccal samples from a total of 189 patients. All patients had at least one actionable variant. The prevalence of patients having 2, 3, or 4 variants was 37.0%, 32.8%, and 12.7%, respectively. Potential alterations to medications were identified using the Clinical Decision Support System. Patients were accepting and the GPs were enthusiastic about the potential of pharmacogenetics to personalize medicine for their patients.

**Conclusion:**

This is the first study in Singapore to demonstrate the feasibility of pharmacogenetic testing in primary care. The high prevalence of genetic variants underscores the potential use of pharmacogenetics in this setting.

**Key messages**

1. Pharmacogenetic testing is feasible in normal Singaporean primary care.
2. There is a high prevalence of potential drug–gene interactions in primary care.
3. Primary care pharmacogenetics needs funding of tests, education, and integrated Clinical Decision Support System.

## Introduction

The study of genetic variation as a factor influencing drug safety, efficacy, and effectiveness has brought about significant breakthroughs in understanding the clinical application of gene–drug interactions to better manage drug therapy. Clinical pharmacogenetics helps healthcare providers use the extensive data gained from clinical trials to implement a drug selection process tailored to an individual and is one of the rapidly advancing applications of precision medicine. It uses variations in an individual’s DNA to identify factors that may impact the clinical effect of a drug on that individual.^1^ A review of multiple studies for cost-effectiveness of pharmacogenomics concluded that this technology has a positive impact on healthcare quality and costs.^2^

One of the challenges of pharmacogenetics is the translation of the large and growing volume of evidence-based information into the multiple clinical settings where prescribing takes place and for the many conditions this technology can be used. This need has been addressed through development of gene–drug guidelines by several international working groups that provide a standardized approach to translating drug–gene association into actionable prescribing recommendation based on the latest collective evidence available. These groups include the Clinical Pharmacogenetics Implementation Consortium (CPIC)^3^ and the Dutch Pharmacogenetics Working Group (DPWG).^4^

The initial efforts to pilot implementation of pharmacogenomics have focused on hospital-based systems, usually with sophisticated electronic medical record systems and highly trained resident experts in the field with access to genetic testing facilities and staff trained in genomics data analysis. Much effort has focused on improving drug therapy within the field of oncology, where several pharmacogenetic tests have been studied for single drugs and a single genetic variation. General practitioners (GPs) do not have the same level of institutional support available, but they are often the source of new drug prescriptions and their patients often require multiple drugs. To impact incidence of adverse drug responses on a national level, it is vital to provide GPs with access to this technology. Recently there has been assessment of pharmacogenetic clinical application in the primary care setting in countries such as Canada, United States, United Kingdom, and the Netherlands. Panels of genes relevant to primary care patients and the cost of testing were investigated in these studies.^5–7^

Although health providers acknowledge that a large opportunity lies in using the technology to optimize prescribing, implementation remains a great challenge in the primary care setting.^8–13^ To maximize the use of pharmacogenetic information for timely and effective prescribing, the presentation of information and medication options must be comprehensible and easily accessible by health providers to facilitate their decision-making process. Limited knowledge and experience on how to interpret the pharmacogenetic data may be another barrier in practical application of the technology. This study was designed to assess the feasibility of collecting buccal samples by GPs at private practices in Singapore within a usual consultation, and the use of a pharmacogenomics-based medical decision support system to guide subsequent drug dosing. It would explore the recruitment capacity of GPs (quantitative), successful DNA collection (quantitative), sample characteristics (quantitative), and preliminary evaluation of GP and patient participants (qualitative).

## Methods

This study is modelled in part on an earlier study that looked at pharmacogenetic testing implemented in a primary healthcare setting in Canada.^7^

We used a prospective cohort study design, with GPs recruiting 189 patients between October 2020 and March 2021. The sample size was calculated on the basis of allele frequencies from a similar primary care study in Canada. Three genes, SCLC01B1, CYP2C9, and CYP2C19 variants with resulting low activity or poor metabolism, were used to generate the sample size using a confidence interval of 95% and an error of 5%. The 3 variants resulted in sample sizes of 31, 59, and 45, respectively. We increased this sample to 150 due to the uncertainty of the mix of ethnicities, and to capture any practical challenges in the recruitment of patients, the testing, and reporting process.

Buccal samples were collected by the GPs and sent to the laboratory, where DNA was extracted, quality assessed, and genotyped. The results were then sent to the Clinical Decision Support System (CDSS) provider to generate reports which were uploaded to the CDSS and linked to the respective GPs’ account. The GPs used the CDSS software to review the patient’s drug therapy for one or more chronic conditions and discuss the findings with the patient. Each patient was followed up for 6 months. The incidence of actionable findings was determined, and the number of cases tracked in which prescribing decisions for patients were altered in response to the pharmacogenetic data coupled with CDSS software.

### Participants

Recruitment of GPs was made by email sent to the 150 GP members of the Singapore Primary Care Research Network (pcRn). A bulletin to members was also posted and personal invitations were made opportunistically. From the 7 practices who responded to this request, 6 were selected based on site feasibility visits. This was the first pcRn multi-practice project and all the selected practices were private which is typical of primary care delivery in Singapore.

The GPs recruited patients at their respective practices. The study population was adults at least 21 years old and being regularly reviewed by their GP for one or more long-term conditions requiring regular medication. The list of included conditions can be found in Supplementary Appendix 1.

### Pharmacogenetic test panel

DNA was extracted from patient samples using the QIAamp DNA mini kit. Samples that passed quality control (QC) were then scheduled for genotyping. The genotypes of 51 biallelic SNPs were determined using Illumina Infinium Global Screening Array (GSA) V2.0 chips and the Illumina iScan System. The Infinium GSA-24 v2.0 BeadChip was selected as it has been widely used in genomic research studies due to its high throughput and low cost. The GSA chip measures over 660,000 markers for multi-ethnic genome-wide content. A limitation of this chip is that it does not provide analysis of HLA alleles and has limited accuracy for assessing copy number variation, both of which are needed to fully utilize known pharmacogenomic markers.

Results for the coded samples were then sent to the provider of the CDSS systems (GenXys, Canada) who provided patient-specific drug dosing guidance based on international guidelines, including CPIC and the DPWG. Actionable findings were determined using CPIC level A and B drug–gene interactions. Only CPIC level A and B gene/drug pairs have sufficient evidence for at least one prescribing action to be recommended. The incidence of actionable findings was tracked using data from the patients pharmacogenetic results mapped against the list of level A and B actionable variants from CPIC.

### Clinical Decision Support System

The CDSS used in this study was provided by GenXys Health Care Systems, using their globally informed guidelines that are integrated into a rules-based system, which delivers drug dosing guidance based on patient genetics (Supplementary Appendix 2). The system as currently deployed is capable of using 58 SNPs, 2 copy number variants (CNV), and 4 HLA alleles to guide dosing for 156 different drugs. In the present study, the genotyping analysis provided information on 46 biallelic SNPs that were used to guide dosing on a subset of 95 drugs. The pharmacogenomic guidance was conveyed to the GPs using a secure link to the GenXys CDSS. The GPs had two 1-h sessions of one-to-one training from the CDSS software developers using dummy patient data prior to receiving and reviewing their patient’s results.

### Study procedures

The GPs identified eligible patients and invited them to participate in the study when they attended their practices for routine clinical care and medication re-supply. The sample size was based on the prevalence of pharmacogenetic variants and the number of physicians taking part. The mean frequency of pharmacogenetic variants detected was calculated. Consent was taken by the GP and a buccal sample was collected using the *ORAcollect.DNA* collection kit at the baseline visit. The GPs discussed the pharmacogenetic findings and medication recommendation with the patient at their next routine visit or when the reports were ready. The software displayed drug options for the conditions selected. During the follow-up period, the GPs self-reported how they used the software. Six months follow-up allowed sufficient time for evidence of drug response and potential adverse drug reactions. The number and nature of each change to medication were recorded.

Patients and doctors were interviewed by members of the local research team. Open-ended questions about the experience were developed following discussion with the research team. The areas explored were the GPs’ and participants’ attitudes to pharmacogenetics, the sample collection process, the results process including the CDSS, the impact on medications, and on education and interpretation.

## Results

Seven GPs from 6 private practices recruited and obtained buccal samples from a total of 189 patients. The cost of sample collection kit, DNA extraction, and genotyping on GSA array was SGD104 (USD75) per sample. Of the 189 samples, 188 (99.5%) were successfully genotyped after first attempt. Only one patient sample (0.5%) had insufficient DNA and a second sample was collected and successfully analysed.

The study population contained 89 females and 100 males with mean age of 63 ± 13 years, ranging from 31 to 100 (Table 1). The majority of the patients presented with 1 and 2 chronic conditions with an occurrence of 28% and 40.9%, respectively. The top 5 chronic conditions among the study population were hypertension, hyperlipidemia, type 2 diabetes mellitus, gout, and osteoarthritis.

**Table 1.** Characteristics of patients recruited for pharmacogenetic testing in Singapore primary care.

Characteristics of patients		*N* (%)
Gender	Male	100 (52.9)
	Female	89 (47.1)
Age	<40	8 (4.2)
	40–49	24 (12.7)
	50–59	42 (22.2)
	60–69	52 (27.5)
	70–79	43 (22.8)
	80–89	19 (10.0)
	>89	1 (0.5)
Number of morbidities	1	54 (28.6)
	2	76 (40.2)
	3	41 (21.7)
	4	9 (4.8)
	5	5 (2.6)
	6	1 (0.5)
Top 10 conditions	Hypertension	141
	Hyperlipidemia	138
	DMT2	55
	Gout	14
	Osteoarthritis	11
	Asthma	8
	Osteoporosis	7
	Anxiety	7
	Dyspepsia	7
	Lower back pain	5

DMT2, type 2 diabetes mellitus.

**Fig. 1.**
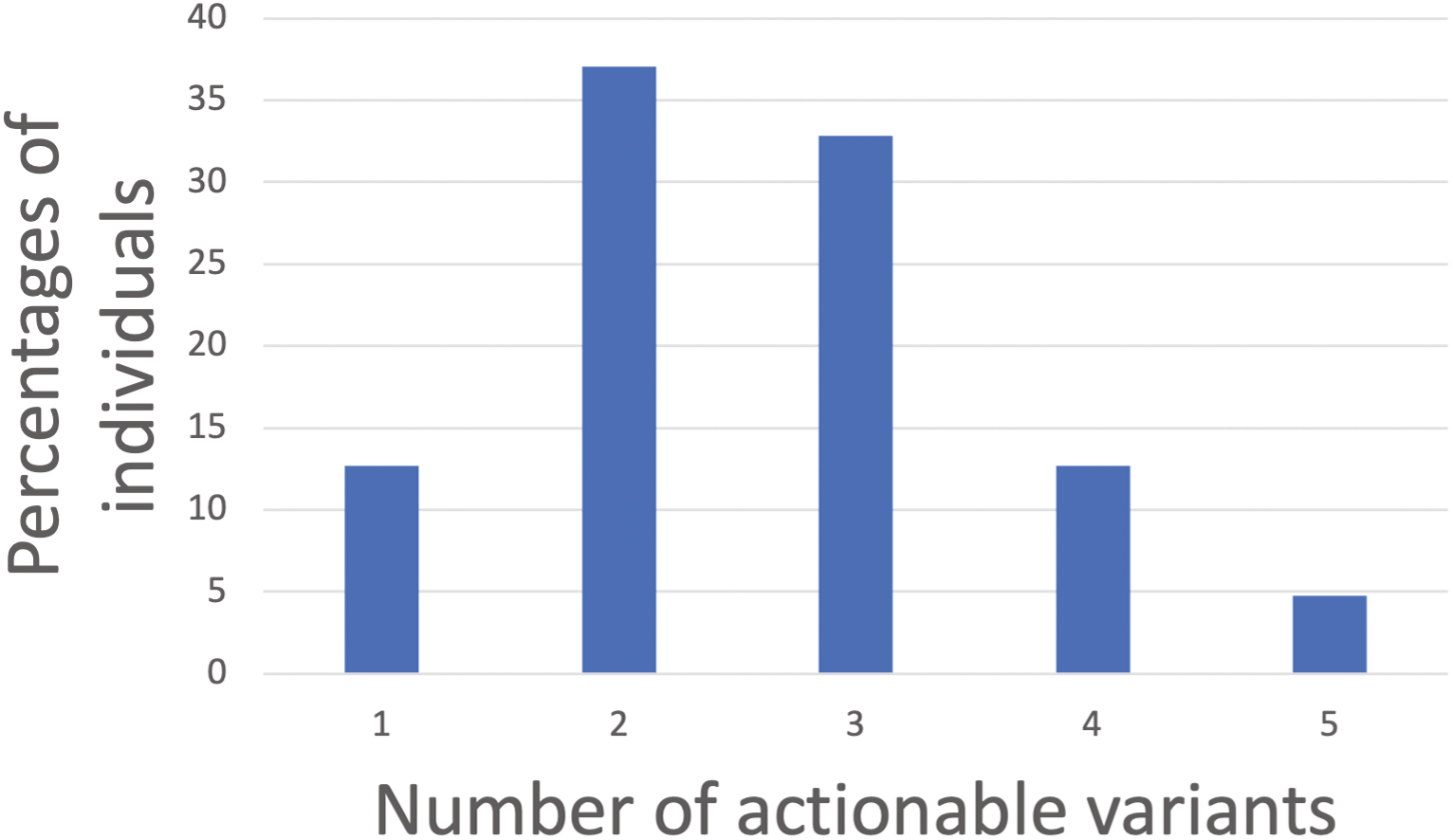
Frequency of actionable pharmacogenetic variants in a Singaporean primary care population (*n* = 189).

All patients had at least one actionable variant; a full list of variants is included (Supplementary Appendix 3). The prevalence of patients having 2, 3, or 4 variants was 37%, 32.8%, and 12.7%, respectively (Fig. 1). The number of potential drugs with CPIC levels A or B evidence of drug–gene interaction associated with these variants was significant (Table 2). Twelve clinically actionable findings (6.3%) were identified by the GPs where 8 of these resulted in either a change or partial change to patient’s current medication while 4 had their medication unchanged due to patient preference in the absence of them experiencing any adverse effects.

**Table 2.** Frequency of alleles, diplotypes, phenotypes, and CPIC A and B drugs with known drug–gene interactions for fully genotyped patients tested in primary care Singapore (*n* = 189).

Gene	Alleles/diplotypes	Phenotype	No. of patients	Frequency of alleles/diplotypes % (95% CI)	CPIC A and B drugs
SLCO1B1 rs4149056	T/T (*1/*1)	Normal function	157	83.1 (78–88)	Atorvastatin Fluvastatin Lovastatin Pitavastatin Pravastatin Rosuvastatin Simvastatin
	T/C (*1/*5)	Decreased function	27	15.3 (9–19)	
	C/C (*5/*5)	Low activity	5	2.3 (0–5)	
VKORC1 rs9923231	G/G	Normal activity	9	4.8 (2–8)	Warfarin
	G/A	Intermediate activity	38	20.1 (14–26)	
	A/A	Low activity	142	75.1 (69–81)	
CYP2C19	*1/*1	Normal metabolizer	86	45.5 (38–53)	Amitriptyline Citalopram Clopidogrel Dexlansoprazole Doxepin Escitalopram Lansoprazole Omeprazole Pantoprazole Voriconazole Lornoxicam
	*1/*2, *1/*3, *2/*17	Intermediate metabolizer	79	41.8 (35–49)	
	*2/*2, *2/*3, *2/*8, *3/*4	Poor metabolizer	20	10.58 (6–15)	
	*1/*17, *17/*17	Ultra-rapid metabolizer	3	1.6 (0–3)	
	Unknown		1		
CYP2C9	*1/*1	Normal metabolizer	171	90.4 (86–95)	Celecoxib Flurbiprofen Fluvastatin Fosphenytoin Ibuprofen Lornoxicam Meloxicam Phenytoin Piroxicam Siponimod Tenoxicam Lenoxicam Warfarin
	*1/*2, *1/*3, *1/*2 (AS: 1.5), *1/*3 (AS: 1.0)	Intermediate metabolizer	18	9.5 (5–14)	
	*3/*3	Poor metabolizer	0		

CPIC level A and B gene/drug pairs have sufficient evidence for at least one prescribing action to be recommended. CPIC level C and D gene/drug pairs are not considered to have adequate evidence or actionability to have prescribing recommendations.

Five GPs and 7 patients participated in qualitative interviews exploring their perceptions and acceptance of pharmacogenetic testing. The open structure to the interviews provided a mixture of negative and positive comments. A selection of representative comments identifies key findings from GP and patient experiences during the study.

The GPs were enthusiastic about the potential of pharmacogenetic to personalize medicine for their patients.

*“It reduces the amount of trial and error that you can have…, it’s probably more scientific, and can tell patients why I’m changing your medicine now” (GP02)*

*“It looks like an emerging discipline or a field. The project was targeted, or at least designed, in a way to include family practice. So, I was interested to see how this emerging field of knowledge can be incorporated into clinical practice in the future.” (GP04)*

*“We are prescribing blind at the moment, so if we have something that’s affordable and can be used at a primary care level, then this is very helpful” (GP05)*

Patients were unfamiliar (*n* = 6) or had an elementary understanding (*n* = 1) of pharmacogenomics; their participation was motivated by a loyalty to their GP or a desire to receive better care.

*“…help me understand what medicines to avoid. And what medicine that will cause adverse effects.” “I may not be aware of some allergies and then the doctors are not aware. So if this information is available to the doctor who attends to me, I don’t mind releasing the information so that I don’t face any side effects and I can get more effective medicine to cure my sickness.” (Patient E085)*

*“I did not know what medications I took whether it’s okay or not, any side effect on me, I do not know. But maybe with this, I hope they can tell me that this is good for me or not good for me.” (Patient E087)*

Obtaining a sample of DNA using a buccal swab was acceptable to both GPs and their patients. GPs described the procedure as *“simple,” “convenient,” “straightforward,”* and readily incorporated into a routine consultation, with such comments as *“I don’t have any problem with the mouth swabs. I think we do even more complicated swabs during COVID times. The mouth swabs were a breeze.” (GP05)* Patients similarly found the test acceptable (*“The procedure is simple. It’s good also…painless.” (Patient C133))* and valued the test not involving venepuncture, *“I was afraid, you know, that they may do …do some blood tests or whatever...” (Patient C146)*

In anticipation of receiving the pharmacogenetic test results, one patient described feeling mildly anxious because of the potential increase in cost of any changes to medication indicated; for others, the time waiting for the test results and the consultation for the pharmacogenetic-facilitated medication review did not provoke anxiety. Patients were generally willing to contribute towards the costs of pharmacogenetic testing (6/7 suggested an acceptable out-of-pocket cost would be in the range SGD20–100 (USD14–72), and one was willing to pay SGD200 (USD144)). Both GPs and patients recognized the necessity of a government subsidy to maximize the uptake and impact of pharmacogenetic.

*“It would be good that it just can be in the mainstream, where it would be part of the treatment process. Where you can deduct from any of the government MediSave and uh, CHAS card* [national health insurance scheme]*.” (GP02)*

Their initial satisfaction with their training was somewhat challenged when beginning to use the CDSS in the clinical setting; they found the navigation of the software not always intuitive (*“I had some problems initially, It doesn’t tell you that you have to go to options to go down to the save mode” (GP02)*) and the volume of results complex (*“The part that is really challenging, and I say wow, this is a long list of drugs” (GP05)*) *“I personally don’t understand the long report. It is too much information there.” (GP01)*

## Discussion

This study provides evidence that pharmacogenomic-guided drug dosing can be applied in a general practice environment in Singapore. We have piloted an implementation process that meets the challenges of obtaining consent, submitting samples for genomic analysis, and using CDSS to guide drug dosing recommendations. This process is able to use genomic data generated using different technologies including microarrays, PCR, and genomic sequencing.

In this first study of primary care pharmacogenetics in Singapore, we have shown that patients, who may benefit from pharmacogenetic information, can be identified, tested, and their results evaluated in normal day-to-day practice. This outcome is similar to studies performed in other jurisdictions.^7,14,15^ The overall high percentage of patients with clinically significant pharmacogenetic variants (>95%) was similar in all 3 prior studies.

Primary care must be an essential venue for pharmacogenetics implementation as the majority of new drug prescriptions come from primary care.^16^ This factor will become even more important as GPs will carry the bulk of increased drug prescribing as the population ages; in Singapore it is estimated that 20% of residents will be aged 65 and above by year 2030. Primary care services are provided by public polyclinics and private general practices in Singapore, with private GPs handling 80% of the primary care services and managing about 60% of chronic patients in the community.^17^ Better management of drug therapy at this level would reduce cost and contribute to improvement in patient outcome. A local study estimated that one-third of hospital admissions due to drug reactions would be prevented if pharmacogenetic test results were available.^18^

The surge in multimorbidity and polypharmacy in primary care is a major concern in a growing global ageing population. Pharmacogenetics may play a meaningful role in optimizing medicines and managing polypharmacy, which becomes even more important in ageing populations. Polypharmacy is often a potential cause of adverse drug events which lead to hospitalization and thereby utilization of healthcare resources.^19^ Fortunately, many of the commonly used drugs used to manage patients with mental health problems, cardiovascular disease, and other common issues treated at the primary care level are included in the established guidelines developed by CPIC and other pharmacogenomic working groups.

Singapore made an early start towards implementation of pharmacogenomics when the Ministry of Health announced in 2013 that genotyping for the HLA-B*1502 allele would be the standard of care prior to the initiation of carbamazepine therapy. This decision followed a very extensive analysis of data from published studies and careful assessment of the economics of implementation in Singapore. However, since that time no other pharmacogenomic tests have been implemented nationally, partly due to the challenge of showing a cost–benefit for other single drug–gene pairs.

In contrast to reactive single-gene assays, the economics of pre-emptive multi-gene testing have been demonstrated in multiple studies when using multiplexed assay platforms that greatly reduce the costs for determining individual genotypes.^20^

This increased volume of genetic information will also require a new approach to gaining regulatory approval for implementing dozens of drug–gene relationships that have reached the highest levels of CPIC evidence. Finally, a robust, secure, and easy-to-use CDSS is needed to help guide GPs to use the pharmacogenetic guidance for delivering the best medical practice for their patients.

Reports on personalized medicine^16^ have emphasized the need to engage many stakeholders, including healthcare providers, biomedical researchers, regulators, drug developers, patient organizations, and patients. To date, the focus has majored on the science and the regulatory environment, and we now need to focus on those stakeholders further down the list, because at the patient level we still have a rudimentary understanding of their concerns and the optimal way to communicate pharmacogenetic results.^21^

### Limitations

This study used Genome Wide Association Studies (GWAS) for obtaining information on patient genotypes and did not provide information on CNV or HLA alleles; therefore, some validated pharmacogenomic biomarkers were not assessed. The medical decision support system used in the study includes the well-characterized drug–gene relationships following CPIC guidelines, which are based on studies conducted mainly in Caucasian populations to inform the clinicians of the phenotype and the dosage modifications indicated. It is possible that there are other alleles, not included in this research study, which are more important in different ethnic groups.

#### Sample size.

This was a feasibility study and so limited in size with 7 family physicians. It required more patients than the 60 calculated for allele frequency. The number was agreed through discussion and consensus. The median and range of patients in feasibility studies in the UK Clinical Research Network is 36 (range 10–300).^22^ The number of patients in this study was higher than the median and is at the upper range for this study methodology.

#### Recruitment bias.

The GPs were the first in Singapore to initiate a pharmacogenetic test for their patients use a broad pharmacogenetic panel. They may not be representative but are the first to give their views on this new process for primary care. The indication from their experience enables the development of larger studies to explore the validity of these findings.

No patients rejected participation according to verbal feedback from the doctors. More than one doctor mentioned they only invited patients whom they thought would be receptive to study participation.

For any new medical procedure to be applied broadly in a medical system, it will need to demonstrate cost-effectiveness in reducing medical expenditures and/or increasing quality and extent of life. Larger studies will be needed to gain definitive information for this critical assessment.

## Conclusion

This is the first study in Singapore to demonstrate the feasibility of pharmacogenetic testing in primary care. The high prevalence of genetic variants underscores the potential use of pharmacogenetics in this setting. GPs identified steps to enable the widescale introduction of pharmacogenetic testing.

## Supplementary material

Supplementary material is available at *Family Practice* online.

cmac124_suppl_Supplementary_Appendix

cmac124_suppl_Supplementary_Checklist

## Data Availability

The data underlying this article cannot be shared publicly due to the privacy of individuals that participated in the study. Data may be shared to individuals in academic organizations with data privacy processes, on reasonable request to the corresponding author.
