# Supplementary material for: Improving prescribing: a feasibility study of pharmacogenetic testing with clinical decision support in primary healthcare in Singapore
Source: Fam Pract. 2022 Nov 23;41(4):477–83. doi: 10.1093/fampra/cmac124 (PMC11324319; doi:10.1093/fampra/cmac124)
Supplement: cmac124_suppl_Supplementary_Appendix [file cmac124_suppl_supplementary_appendix.pdf]

Appendix 1 Long term conditions included in eligibility checklist.

Body system	Conditions
Cardiovascular	Atrial Fibrillation (anticoagulation, rate control)
	Heart Failure (chronic, fluid retention)
	Hyperlipidemia
	Hypertension
	Peripheral Arterial Disease (symptomatic)
	Post Myocardial Infarction
Musculoskeletal	Gout (acute, chronic)
	Osteoarthritis
	Osteoporosis
	Rheumatoid Arthritis
Pain	Fibromyalgia
	Lower Back Pain
	Neuropathic Pain
	Trigeminal Neuralgia
Mental Health	Anxiety (generalized anxiety disorder, social anxiety disorder)
	Bipolar 1 Disorder
	Depression, Schizophrenia
Neurological	Epilepsy
	Migraine (treatment, prophylaxis)
Respiratory	Asthma
	Chronic Obstructive Pulmonary Disease (acute exacerbation, stable)
Endocrine	Diabetes Mellitus Type 2
Gastrointestinal	Dyspepsia
	Prevention of NSAID-Induced Ulcers

Medication Options

Conditions	Condition Details	Medication Options	Expand all

Depression

Treatment Strategy
Monotherapy

Sort Antidepressants By
Default

Condition-Specific Questions
None of the above

Chronic Conditions/Diagnoses
None of the above

Age (years)
32

Kidney Function - eGFR (ml/min)
Moderate Kidney Impairment: 50

Liver Impairment (Child-Pugh Scale)
No liver disease

Genetics - CYP2C19

Preferred Therapy Options

Second-generation antidepressant

See below for side effect profile key

Duration of medication trial: improvement with ongoing antidepressant monotherapy is unlikely if there has been a lack of response after 8 weeks, although continued improvement may be seen, with declining probability, for up to 3 months (Henssler et al. 2018).

Citalopram (Antidepressant, SSRI) $$

Citalopram (Antidepressant, SSRI) $$

**Initial Dose**	10 mg PO once daily
**Titration**	Increase daily dose at 1 week intervals as needed and tolerated (in those who are not CYP2C19 poor metabolizers)
**Maximum Dose**	20 mg PO per day
**Side Effects**	Ach -, GI ++, Sed -, Ins/Agi ++, SexDys ++, OrthHyp -, Weight -, QTc +, LD+, ADS Moderate risk
**Brands**	Celexa, generics

Initial dose reduced by 50% of standard dose (per CPIC guideline to consider 50% reduction in starting dose, moderate recommendation; or select alternative drug not predominantly metabolized by CYP2C19) and maximum dose reduced (as per product monograph) due to CYP2C19 poor metabolizer status

Escitalopram (An

Fluoxetine (Antid

Fluvoxamine (Ant

Paroxetine IR (An

Paroxetine CR (A

Sertraline (Antide

Desvenlafaxine (Antidepressant, SNRI) $$$

Duloxetine (Antidepressant, SNRI) $$

Add Condition | Generate Medication Options | Generate Report

brought to you by GenXys

Appendix 3. SNP Frequencies from 189 patients recruited in Singaporean General Practice				
**Gene**	**Alleles/Diplotypes**	**Phenotype**	**No. of patients**	**Frequency of Alleles/ Diplotypes (95%CI)**
**ABCG2** rs223114	G/G	Typical response	93	49.2 (42 to 56)
	G/T	Increased response	72	38.10 (31 to 45)
	T/T	Increased response	24	12.7 (8 to 17)
**ADRB2** rs1042713	G/G	Typical response	33	17.4 (12 to 23)
	A/G	Typical response	94	49.8 (43 to 57)
	A/A	Reduced response	62	32.8 (26 to 40)
**ANKK1** rs1800497	A/A	Increased risk of weight gain	23	12.2 (8 to 17)
	A/G	Increased risk of weight gain	88	46.5 (40 to 54)
	G/G	Increased risk of tardive dyskinesia	78	41.3 (34 to 48)
**CYP2C19**	*1/*1	Normal metabolizer	86	45.5 (38-53)
	*1/*2,*1/*3,*2/*17	Intermediate metabolizer	79	41.8 (35 to 49)
	*2/*2,*2/*3,*2/*8,*3/*4	Poor metabolizer	20	10.58 (6 to 15)
	*1/*17,*17/*17	Ultra-rapid metabolizer	3	1.6 (0 to 3)
	Unknown		1	
**CYP2C9**	*1/*1,	Normal metabolizer	171	90.4 (86 to 95)
	*1/*2,*1/*3 *1/*2 (AS: 1.5)*1/*3 (AS: 1.0)	Intermediate metabolizer	18	9.5 (5 to 14)
	*3/*3	Poor metabolizer	0	
**CYP3A5**	*1/*1	Normal metabolizer	14	7.4 (11 to 4)
**CYP3A5**	*1/*3	Intermediate metabolizer	74	39.2 (46 to 32)
**CYP3A5**	*3/*3	Poor metabolizer	101	53.4 (60 to 46)
**DPYD**	*1/*1	Normal metabolizer	189	100
**DPYD**	*1/*2, *1/A	Intermediate metabolizer	0	0
	*2/*2, *2/A, A/A	Poor metabolizer	0	0
**DRD2** rs1799978	C/C	Reduced response	8	4.2 (1 to 7)
	C/T	Typical response	58	30.7 (24 to 37)
	T/T	Typical response	123	65.1 (59 to 72)
**Factor II** rs1799963	G/G	Typical risk of adverse drug reactions	188	99.5 (98 to 100)
	G/A	Increased risk of adverse drug reactions	0	0
	A/A	Increased risk of adverse drug reactions	0	0
	Unknown	Unknown	1	0.5
	C/C	Typical risk of adverse drug reactions	189	100

**Factor V** rs6025	C/T	Increased risk of adverse drug reactions	0	0
	T/T	Increased risk of adverse drug reactions	0	0
**FKBP5** rs4713916	G/G	Typical response	105	55. (48 to 63
	A/G	Increased response	74	39.2 (32 to 46
	A/A	Increased response	10	5.3 (2 to 8)
**GNB3** rs5443	C/C	Typical response	44	23.3 (17 to 29)
	C/T	Typical response	80	42.3 (35 to 49)
	T/T	Increased response	65	34.4 (28 to 41)
**GRIK4** rs1954787	T/T	Reduced response	6	3.2 (6 to 28)
	C/T	Reduced response	46	24.3 (18 to 30)
	C/C	Typical response	137	72.5 (66 to 79)
**HTR2A** rs7997012	G/G	Typical response	94	49.8 (42 to 57)
	A/G	Increased response	79	41.8 (35 to 49)
	A/A	Increased response	16	8.4 (4 to 12)
**HTR2C** rs1414334	G/G	Typical risk of adverse drug reactions	184	97.3 (95 to 99)
	C/G	Increased risk of adverse drug reactions	2	1.1 (-0.4-2.5)
	C/C	Increased risk of adverse drug reactions	3	1.6 (-0.2-4)
**IFNL3** rs12979860	C/C	Typical response	162	85.7 (81 to 90)
	C/T	Reduced response	25	13.2 (8 to 18)
	T/T	Reduced response	2	1.1 (-0.4 to 2.5)
**KCNIP4** rs1495509	C/C	Increased risk of adverse drug reactions	34	18.0 (12.5-23)
	C/T	Increased risk of adverse drug reactions	101	53.4 (46.3-60)
	T/T	Typical risk of adverse drug reactions	54	28.6 (22 to 35)
**MC4R** rs489693	C/C	Typical risk of adverse drug reactions	117	61.9 (55 to 69)
	C/A	Typical risk of adverse drug reactions	64	33.9 (27 to 41)
	A/A	Increased risk of adverse drug reactions	8	4.2 (1 to 7)
**NUDT15** rs116855232	C/C	Normal metabolizer	158	83.6 (78 to 89)
	C/T	Intermediate metabolizer	30	15.9 (21 to 11)
	T/T	Poor metabolizer	1	0.5 (-0.5 to 1.56)

**OPRM1** rs1799971	G/G	Reduced response	22	11.6 (7 to 16)
	A/G	Reduced response	90	47.6 (40 to 55)
	A/A	Typical response	77	40.7 (34 to 48)
**PTGS1** rs10306114	A/A	Typical response	188	99.5 (98 to 100)
	A/G	Reduced response	1	0.5 (-0.5 to 1)
	G/G	Reduced response	0	0
**SLCO1B1** rs4149056	T/T (*1/*1)	Normal function	157	83.1(78 to 88)
	T/C (*1/*5)	Decreased function	27	15.3 (9 to 19)
	C/C (*5/*5)	Low activity	5	2.3 (0-5)
**TCF7L2** rs7903146	C/C	Typical response	178	94.2 (91 to 97)
	C/T	Reduced response	11	5.8 (2 to 9)
	T/T	Reduced response	0	0.00%
**TNF-alpha** rs1800629	G/G	Typical response	157	83.1 (78 to 88)
	A/G	Reduced response	31	16.4 (11 to 21)
	A/A	Reduced response	1	0.5 (-0.51 to 1.56)
**TPMT**	*1/*1	Normal Metabolizer	181	95.8 (93 to 99)
	*1/*3	Intermediate Metabolizer	7	3.7 (1 to 6)
	*3/*3	Poor Metabolizer	0	0
	Unknown	Unknown	1	0.5
**VKORC1** rs9923231	G/G	Normal activity	9	4.8 (2 to 8)
	G/A	Intermediate activity	38	20.1 (14 to 26)
	A/A	Low activity	142	75.1 (69 to 81)

Appendix 3. **Full list of alleles, diplotypes, phenotypes and frequencies for genotyped patients tested in primary care Singapore (n=189)**
